# Supplementary material for: Dynamical coupling between a nuclear spin ensemble and electromechanical phonons
Source: Nat Commun. 2018 Aug 28;9:2993. doi: 10.1038/s41467-018-05463-3 (PMC6113237; doi:10.1038/s41467-018-05463-3)
Supplement: Supplementary file 1 — Supplementary Information [file 41467_2018_5463_MOESM1_ESM.pdf]

# Supplemental Information
# Dynamical coupling between a nuclear spin ensemble and electromechanical phonons

Okazaki et al.

## Supplementary Note 1. DERIVATION OF THE QUADRUPOLE HAMILTONIAN

In this section, the derivation of the quadrupole Hamiltonian in Eq. (1) is described along with the determination of the quadrupole coupling constants. The quadrupole interaction at the relevant nuclei's position is governed by an electric field gradient, which is associated with changes in neighboring atomic positions and in their chemical bonds that is caused by finite lattice strain. The resulting energy shift for a nucleus can then be described by the quadrupole Hamiltonian[1, 2]:

$$\hat{H}_{\mathrm{Q}} = \frac{eZ}{6I(2I-1)} \sum_{i,j} V_{ij} \left\{ \frac{3}{2} \left( \hat{I}_i \hat{I}_j + \hat{I}_j \hat{I}_i \right) + \delta_{i,j} I^2 \right\}, \tag{1}$$

where $Z$ is the quadrupole moment, $\delta_{i,j}$ is the Kronecker delta, and $\hat{I}_i$ with $i, j = x, y, z$ is $i$th component of the Pauli spin operator. Here $V_{ij}$ is the electric field gradient tensor, which relates to the elastic strain in the host crystal:

$$V_{ij} = \sum_{i,j} S_{ijkl} \varepsilon_{kl}, \tag{2}$$

where $S_{ijkl}$ is the fourth-rank tensor that relates the electric field gradient at the nuclei's position to the elastic strain field with the strain tensor $\varepsilon_{ij}$.

For a nucleus with $I = 3/2$, the quadrupole Hamiltonian can be reduced to a $4 \times 4$ matrix of the form [3]:

$$\hat{H}_{\mathrm{Q}} = \frac{eZ}{12} \begin{bmatrix} V_{2,0} & V_{2,-1} & V_{2,-2} & 0 \\ V_{2,1} & -V_{2,0} & 0 & V_{2,-2} \\ V_{2,2} & 0 & -V_{2,0} & V_{2,-1} \\ 0 & V_{2,2} & V_{2,1} & V_{2,0} \end{bmatrix}, \tag{3}$$

where $V_{2,0}$, $V_{2,\pm1}$, and $V_{2,\pm2}$ are defined as:

$$V_{2,0} = 3V_{zz}, \tag{4a}$$
$$V_{2,\pm1} = \mp 2\sqrt{3} \left( V_{xz} \pm V_{yz} \right), \tag{4b}$$
$$V_{2,\pm2} = \sqrt{3} \left( V_{xx} - V_{yy} \right) \pm i2\sqrt{3} V_{xy}. \tag{4c}$$

In addition, by taking account of $\mathrm{T_d}$-tetrahedral symmetry of GaAs, Supplementary Equation 2 can be reduced to

$$V_{xx} = \frac{1}{2} S_{11} \left\{ 2\varepsilon_{xx} - (\varepsilon_{yy} + \varepsilon_{zz}) \right\}, \tag{5a}$$
$$V_{yy} = \frac{1}{2} S_{11} \left\{ 2\varepsilon_{yy} - (\varepsilon_{xx} + \varepsilon_{zz}) \right\}, \tag{5b}$$
$$V_{zz} = \frac{1}{2} S_{11} \left\{ 2\varepsilon_{zz} - (\varepsilon_{xx} + \varepsilon_{yy}) \right\}, \tag{5c}$$
$$V_{yz} = 2S_{44} \varepsilon_{yz}, \tag{5d}$$
$$V_{xz} = 2S_{44} \varepsilon_{xz}, \tag{5e}$$
$$V_{xy} = 2S_{44} \varepsilon_{xy}, \tag{5f}$$

where $S_{11}$ and $S_{44}$ are the two independent components of $S_{ijkl}$ in Voigt notation [1, 2]. Note that Supplementary Equation 5 is valid in the coordinate system: X//[010], Y//[001], Z//[100].

Quantification of Supplementary Equations 3 through 5 needs numerical values for the strain tensor components associated with the fundamental flexural motion of the resonator. Hence we perform a finite element method simulation of the motional strain using a commercially available simulator (ANSYS). A doubly-clamped mechanical resonator with a length of 50 $\mu$m, width of 6 $\mu$m, and a thickness of 1 $\mu$m along with the under-etched substrate over which the resonator is suspended are modelled in the simulation as shown in Supplementary Figure 2a. For simplicity, the detailed layer structure consisting the heterowafer as well as thin Schottky electrodes fabricated on the mechanical element are omitted, instead the resonator is imply modelled as pure GaAs. Supplementary Figure 2b shows the six independent components of the strain tensor associated with the fundamental flexural motion. To quantify the quadrupole Hamiltonian at the clamping point, the numerical values are extracted at the point indicated by $\varepsilon$ in Supplementary Figure 2a, where the strain tensor is normalized by the displacement $x$ of the fundamental mode

measured at the center of the beam (indicated by $x$ in Supplementary Figure 2a) which yields:

$$\begin{bmatrix} \varepsilon_{xx} \\ \varepsilon_{yy} \\ \varepsilon_{zz} \\ \varepsilon_{yz} \\ \varepsilon_{xz} \\ \varepsilon_{xy} \end{bmatrix} = \begin{bmatrix} -899 \\ -908 \\ 812 \\ 24 \\ 21 \\ -1562 \end{bmatrix} x. \tag{6}$$

From these numerical values, it can be confirmed that the terms $V_{2,\pm 1}$ and $(V_{xx} - V_{yy})$ in Supplementary Equation 4 are negligibly small compared to the others. As a result, the quadrupole operator normalized by the mechanical displacement $x$, i.e. $\hat{J}_{\mathrm{Q}} = \hat{H}_{\mathrm{Q}}/x$ can be approximated as

$$\begin{aligned} \hat{J}_{\mathrm{Q}} &\approx \begin{bmatrix} A_1 & 0 & i2\sqrt{3}A_2 & 0 \\ 0 & -A_1 & 0 & i2\sqrt{3}A_2 \\ -i2\sqrt{3}A_2 & 0 & -A_1 & 0 \\ 0 & -i2\sqrt{3}A_2 & 0 & A_1 \end{bmatrix} \\ &= A_1 \left[ 3\hat{I}_z^2 - I(I+1) \right] /3 + iA_2 \left[ \hat{I}_+^2 - \hat{I}_-^2 \right], \end{aligned} \tag{7}$$

where $\hat{I}_+$ and $\hat{I}_-$ are the spin ladder operators, and

$$A_1 = \frac{eZ}{8} S_{11} \left( 2\varepsilon_{zz} - \varepsilon_{xx} - \varepsilon_{yy} \right) /x, \tag{8a}$$

$$A_2 = -\frac{eZ}{6} S_{44} \varepsilon_{xy}/x. \tag{8b}$$

For $^{75}$As in GaAs, $Z = 2.9 \times 10^{-29}$ m$^2$, $S_{11} = 4.0 \times 10^{22}$ V m$^{-2}$, and $S_{44} = 8.0 \times 10^{22}$ V m$^{-2}$ are taken from Refs. [1, 2]. Finally substituting Supplementary Equation 6 into 8 yields $A_1 = 0.49$ meV m$^{-1}$, and $A_2 = -0.6$ meV m$^{-1}$. Similarly, for $^{69}$Ga in GaAs, $Z = 1.9 \times 10^{-29}$ m$^2$, $S_{11} = 2.7 \times 10^{22}$ V m$^{-2}$, $S_{44} = 2.8 \times 10^{22}$ V m$^{-2}$, $A_1 = 0.16$ meV m$^{-1}$, and $A_2 = -0.14$ meV m$^{-1}$ are determined.

## Supplementary References


[1] R. K. Sundfors, Phys. Rev. **177**, 1221 (1969).
[2] R. K. Sundfors, Phys. Rev. B **10**, 4244 (1974).
[3] M. Ono, J. Ishihara, G. Sato, S. Matsuzaka, Y. Ohno, and H. Ohno, Phys. Rev. B **89**, 115308 (2014).

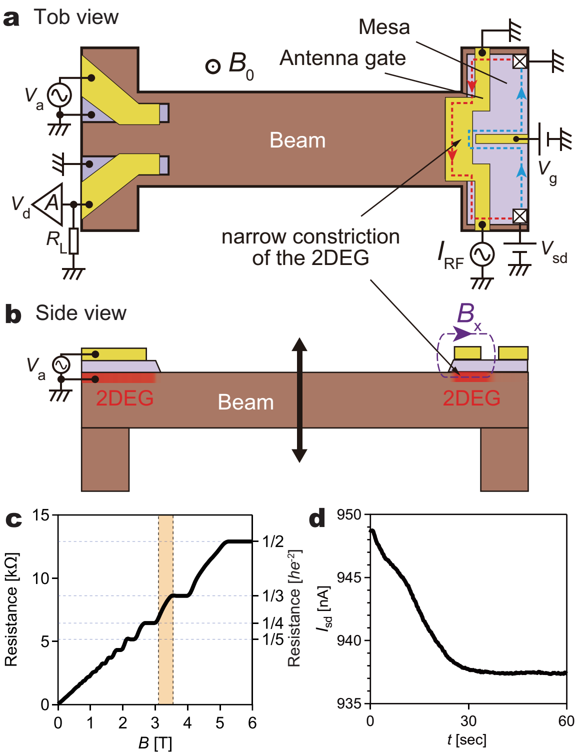


Supplementary Figure 1. **Experimental setup for resistively detected NMR**. **a** and **b**, Schematic of the device structure and the measurement setup from top (**a**) and side (**b**). The two counter propagating edge channels (red and blue dashed lines) are squeezed to form a narrow constriction of approximately 1.5 $\mu$m in width. **c**, Two-terminal resistance of the 2DEG measured as a function of $B_0$ with $V_g = 0$ V, where the series resistance from wires and filters is subtracted. The plot shows the integer quantum Hall plateaus at $h/\nu e^2$ with integer values $\nu$, with some indicated in the right axis, where $h$ and $e$ are the Planck constant and elementary charge, respectively. The orange area corresponds to $3.1 < B_0 < 3.55$ T, where the resistively detected NMR is performed. **d**, The time evolution of $I_{sd}$, after the abrupt application of a constant source-drain voltage $V_{sd} = 13.2$ mV at $t = 0$, exhibiting a slow decrease for the first 30 seconds due to dynamic nuclear polarization.

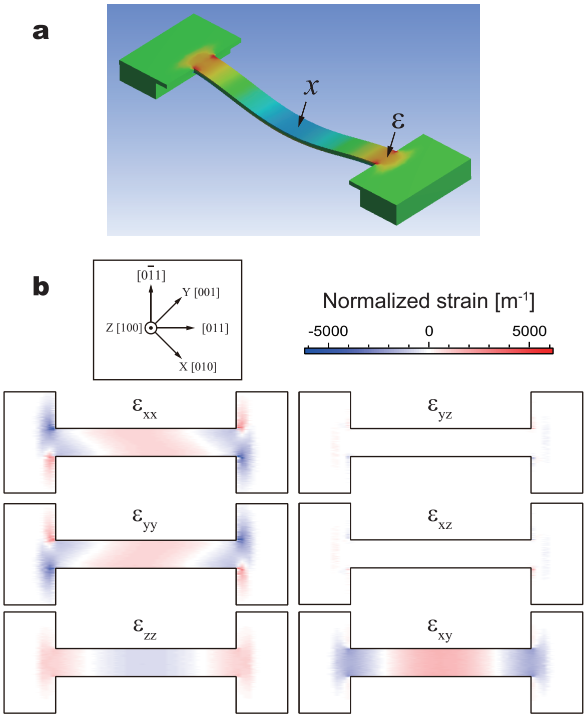


Supplementary Figure 2. **Numerical simulation of the motional strain**. **a**, Three-dimensional model of the doubly-clamped electromechanical resonator used in the numerical simulation. **b**, The color plots show the six independent strain tensor components associated with the fundamental flexural motion. The strain components are normalized by the displacement of the resonator measured at the center of the beam [$x$ in **a**], with unit of [m$^{-1}$]. The X-axis (Y-axis) is defined along the [010] ([001]) orientation of the GaAs crystal while the resonator is fabricated along [011] as depicted in the upper schematic. From the simulation numerical values for the strain tensor at the right clamping point, indicated by $\varepsilon$, are extracted.

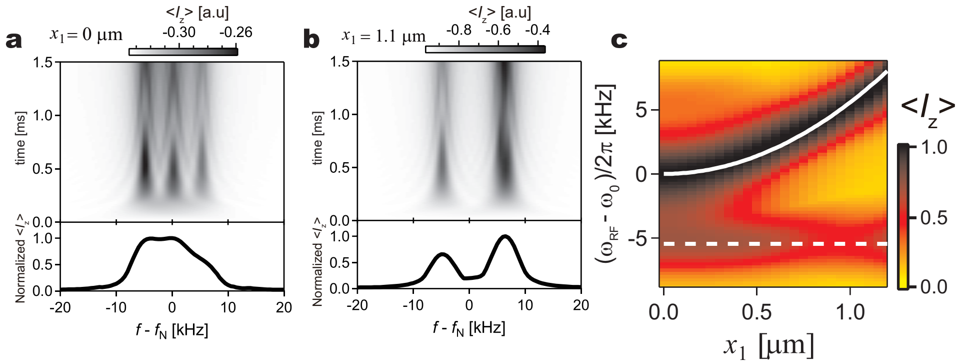


Supplementary Figure 3. **Numerical simulation of the NMR spectra under driven electromechanical strain**. **a**,**b**, The time-evolution of $\langle I_z \rangle$ for $^{75}$As nuclei with $x_1 = 0$ $\mu$m (**a**) and $x_1 = 1.1$ $\mu$m (**b**). The upper panels show the temporal evolution of $\langle I_z \rangle$ and the lower panels show $\langle I_z \rangle$ normalized by the oscillation amplitude. **c**, A calculated NMR contour plot showing $\langle I_z \rangle$ as a function of $x_1$. The solid (dashed) line shows the theoretical frequency shift derived from the Floquet perturbation theory for the transition $|1/2\rangle \leftrightarrow |-1/2\rangle$ ($|-3/2\rangle \leftrightarrow |-1/2\rangle$).
